# Acetoacetate, a ketone body, attenuates neuronal bursts in acutely-induced epileptiform slices of the mouse hippocampus

**DOI:** 10.3389/fncel.2025.1551700

**Published:** 2025-03-05

**Authors:** Hao Wen, Nagisa Sada, Tsuyoshi Inoue

**Affiliations:** Department of Biophysical Chemistry, Graduate School of Medicine, Dentistry and Pharmaceutical Sciences, Okayama University, Okayama, Japan

**Keywords:** epilepsy, ketone body, ketogenic diet, hippocampus, slice physiology, patch-clamp recording

## Abstract

The ketogenic diet increases ketone bodies (β-hydroxybutyrate and acetoacetate) in the brain, and ameliorates epileptic seizures *in vivo*. However, ketone bodies exert weak or no effects on electrical activity in rodent hippocampal slices. Especially, it remains unclear what kinds of conditions are required to strengthen the actions of ketone bodies in hippocampal slices. In the present study, we examined the effects of acetoacetate on hippocampal pyramidal cells in normal slices and epileptiform slices of mice. By using patch-clamp recordings from CA1 pyramidal cells, we first confirmed that acetoacetate did not change the membrane potentials and intrinsic properties of pyramidal cells in normal slices. However, we found that acetoacetate weakened spontaneous epileptiform bursts in pyramidal cells of epileptiform slices, which were acutely induced by applying convulsants to normal slices. Interestingly, acetoacetate did not change the frequency of the epileptiform bursts, but attenuated individual epileptiform bursts. We finally examined the effects of acetoacetate on excitatory synaptic barrages during epileptiform activity, and found that acetoacetate weakened epileptiform bursts by reducing synchronous synaptic inputs. These results show that acetoacetate attenuated neuronal bursts in epileptiform slices, but did not affect neuronal activity in normal slices, which leads to seizure-selective actions of ketone bodies.

## Introduction

Epilepsy is one of the most common neurological disorders in the world (Ngugi et al., 2010), but currently-used antiepileptic drugs are not effective for approximately 30% of epileptic patients (Kwan and Brodie, 2000; Chen et al., 2018). It is well known that the ketogenic diet is effective for the drug-resistant epilepsy (Neal et al., 2008, 2009). Epilepsy treatment using the ketogenic diet was originally developed in the 1920s (Wilder, 1921), and its modified version using a medium-chain triglyceride was developed in the 1970s (Huttenlocher et al., 1971). These ketogenic diets consist of high-fat and low-carbohydrate, which increase ketone bodies (β-hydroxybutyrate and acetoacetate) and mildly decrease glucose levels in epileptic patients (Huttenlocher, 1976). These two metabolic changes are presumed to suppress epileptic seizures (reviewed in Rho, 2017).

Previous studies have reported the molecules that electrically regulate the antiseizure actions of the ketogenic diet (reviewed in Sada and Inoue, 2018). Regarding decreases in glucose, the ketogenic diet suppresses seizures via adenosine A_1_ receptors (Masino et al., 2011), which is due to decreases in glucose (Kawamura et al., 2010). The ketogenic diet also decreases lactate levels in the brain, which consequently hyperpolarizes neurons and suppresses seizures (Sada et al., 2015). The inhibition of lactate dehydrogenase also decreases lactate levels and suppresses seizures (Sada et al., 2020). Regarding increases in ketone bodies, ketone bodies open ATP-sensitive K^+^ channels (K_ATP_ channels) and reduce the firing rate of neurons (Ma et al., 2007; Tanner et al., 2011). Acetoacetate inhibits vesicular glutamate transporters (VGLUTs) and reduces miniature excitatory postsynaptic currents (EPSCs) in hippocampal slices (Juge et al., 2010). Acetoacetate also inhibits voltage-dependent Ca^2+^ channels (VDCCs) and reduces EPSCs in hippocampal slices (Kadowaki et al., 2017).

Electrophysiology using hippocampal slices has been used in mechanistic studies at the synaptic and network levels. However, previous studies have reported that ketone bodies exert weak or no effects on hippocampal slices obtained from normal rodents, which cannot fully explain the antiseizure actions of the ketogenic diet *in vivo* (reviewed in Kawamura et al., 2016). For example, ketone bodies reduce the firing rate in neurons via K_ATP_ channels by only 10% (Ma et al., 2007). Acetoacetate reduces miniature EPSCs via VGLUTs by only 25% (Juge et al., 2010), and inhibits VDCCs by only 20% (Kadowaki et al., 2017). Furthermore, several studies have reported that ketone bodies have no effects on synaptic transmission and long-term potentiation in hippocampal slices (Thio et al., 2000; Kimura et al., 2012; Youssef, 2015), and chronic exposure to 10 mM β-hydroxybutyrate also have no effects on stimulus-induced discharges in organotypic hippocampal slices (Samoilova et al., 2010). Therefore, it is important to find what kinds of conditions are required for ketone bodies to suppress electrical activity in hippocampal slices, in order to fill the gap between brain slices *in vitro* and seizures *in vivo*.

## Materials and methods

### Animals and slice preparation

Experiments were performed using ICR mice (postnatal days 17–35) for patch-clamp recordings *in vitro* from hippocampal slices. All experimental procedures were approved by the Animal Research Committee at Okayama University. Slice preparation and recordings were performed as previously described with minor modifications (Sada et al., 2015; Kadowaki et al., 2017). Mice were anesthetized with isoflurane and killed by decapitation, and the brain was removed and placed in an ice-cold dissecting solution (in mM): 234 sucrose, 2.5 KCl, 1.25 NaH_2_PO_4_, 25 NaHCO_3_, 10 MgSO_4_, 12 glucose, and 0.5 CaCl_2_. Transverse hippocampal slices (300 μm thick) were made using a vibratome by horizontal cutting of the ventral hippocampus. The slices were then incubated at 32°C for 30 min in artificial cerebrospinal fluid (ACSF) (in mM): 125 NaCl, 2.5 KCl, 1.25 NaH_2_PO_4_, 1.2 MgSO_4_, 25 NaHCO_3_, 12 glucose, and 2.5 CaCl_2_, bubbled with 95% O_2_ and 5% CO_2_. The slices were then placed at room temperature until before recordings.

### Patch-clamp recording

**Figure 1:**
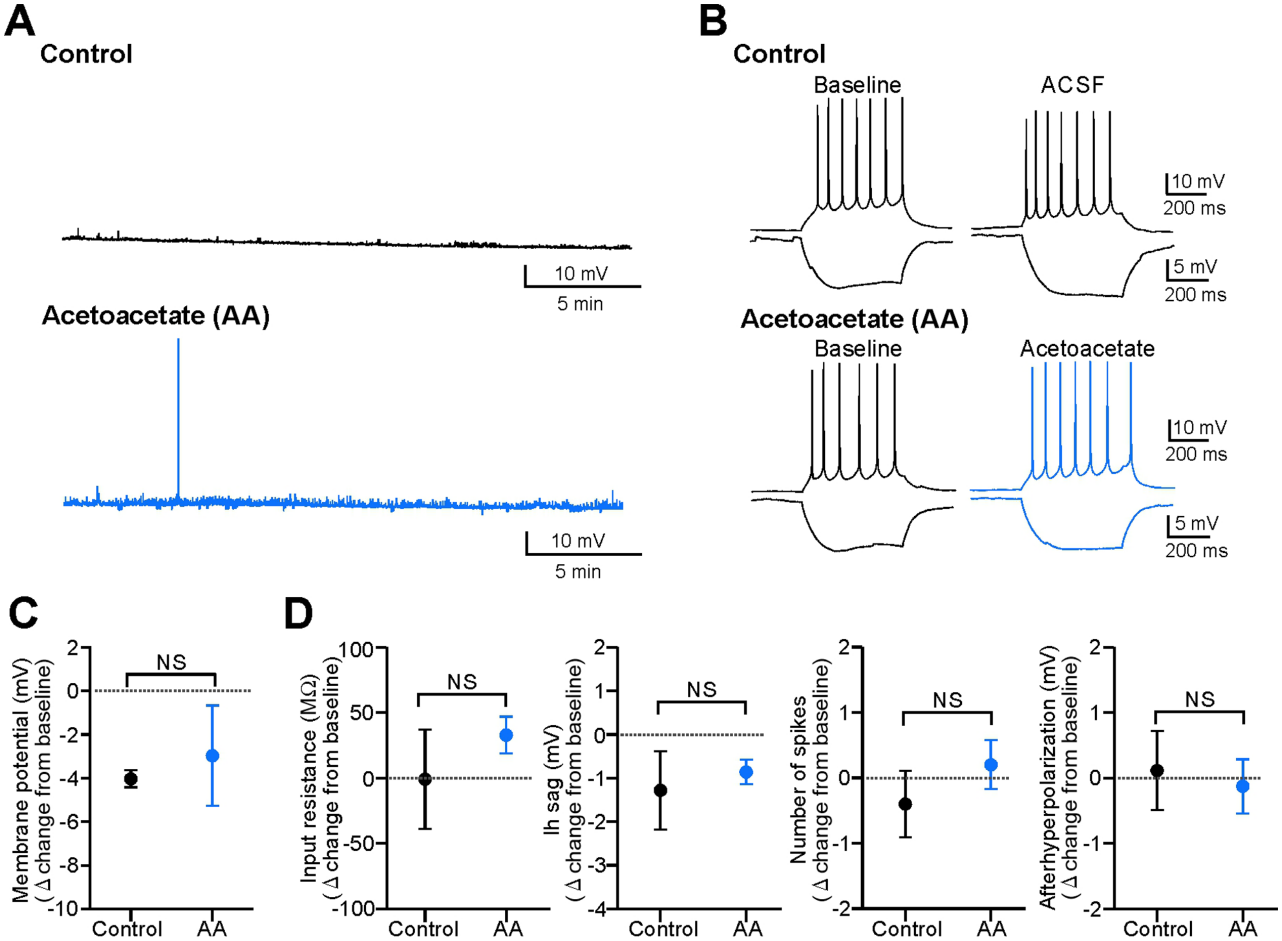
Acetoacetate does not change electrical properties in hippocampal pyramidal cells of normal slices. **(A)** Membrane potentials in pyramidal cells, changed by a 20-min recording with no reagents for the control (*Control*) or by a 20-min application of 10 mM sodium acetoacetate (*Acetoacetate*, abbreviated as *AA*). Membrane potentials were set to −70 mV at the baseline, and reagents were then applied. **(B)** Membrane intrinsic properties in pyramidal cells, changed by a 20-min recording with no reagents for the control (*Control*) or by a 20-min application of 10 mM sodium acetoacetate (*Acetoacetate*). Intrinsic properties were measured by a 500-ms injection of ±50 pA from −60 mV. **(C)** Summary data from **(A)**, showing the changes in membrane potentials from the baseline in the control group (*n* = 5) and acetoacetate-treated group (*n* = 5). **(D)** Summary data from **(B)**, showing the changes in membrane intrinsic properties (input resistance and Ih sag measured by a −50 pA injection, and the number of spikes and afterhyperpolarization measured by a +50 pA injection) from the baseline in the control group (*n* = 5) and acetoacetate-treated group (*n* = 5). NS, not significant (Mann–Whitney test).

**Figure 2:**
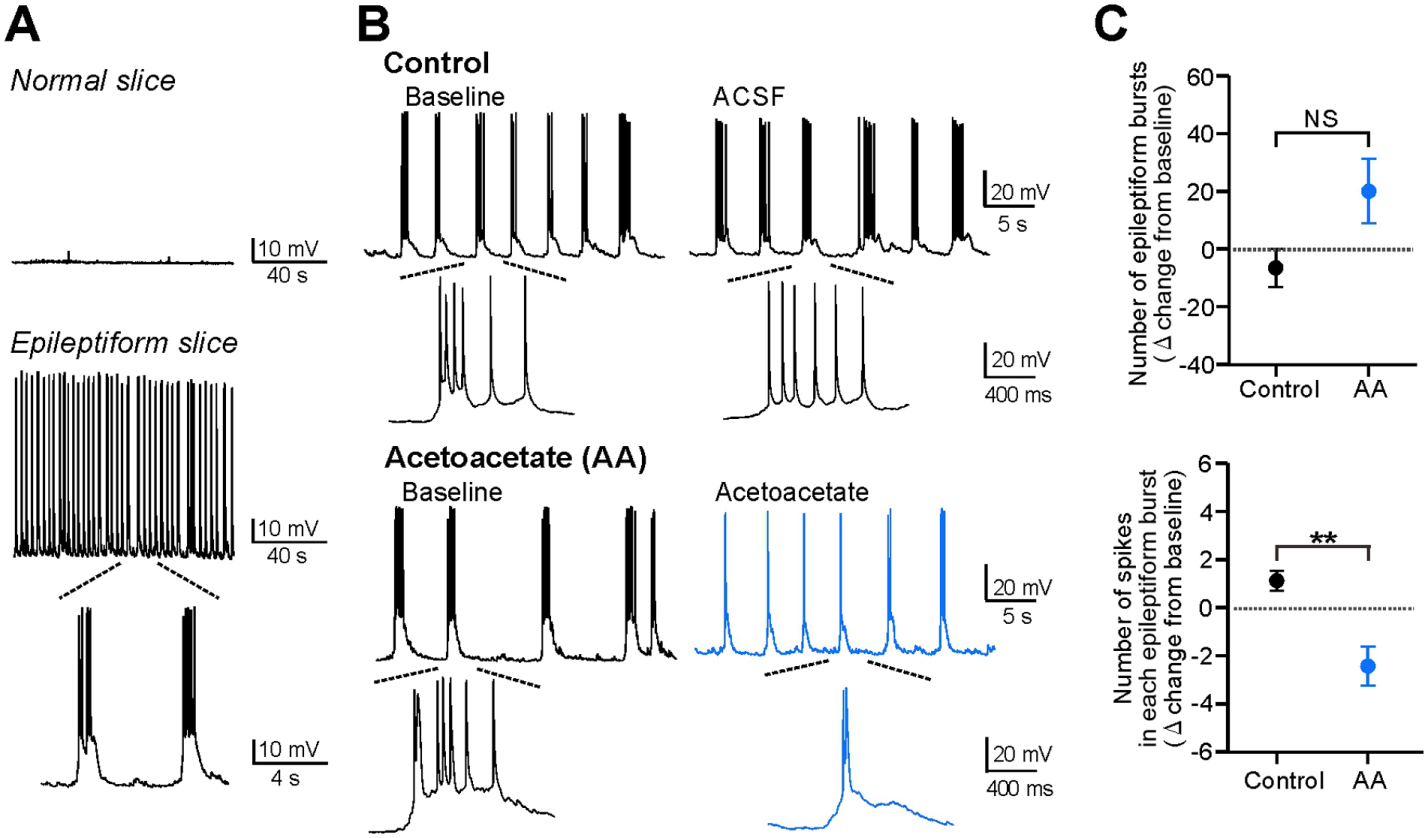
Acetoacetate attenuates individual epileptiform bursts in hippocampal pyramidal cells of epileptiform slices. **(A)** Silent membrane potentials in normal slices (*upper*) and spontaneous bursts in epileptiform slices (*lower*), measured by current-clamp recordings from CA1 pyramidal cells. Spontaneous epileptiform bursts were induced by the bath-application of the following blocker cocktails, the K^+^ channel blockers TEA-Cl and CsCl, the GABA_A_ receptor blocker picrotoxin, and the GABA_B_ receptor blocker CGP-55845. **(B)** Epileptiform bursts in pyramidal cells, changed by a 20-min recording with no reagents for the control (*Control*) or by a 20-min application of 10 mM sodium acetoacetate (*Acetoacetate*). Epileptiform bursts were measured at a membrane potential of −75 mV. **(C)** Summary data from **(B)**, showing the changes in the number of epileptiform bursts for 5 min (*upper*) and the number of spikes in each epileptiform burst (*lower*) from the baseline in the control group (*n* = 6) and acetoacetate-treated group (*n* = 6). Epileptiform bursts were evaluated from 5-min recordings at the baseline and 15–20 min after the application of acetoacetate. ^**^p < 0.01; NS, not significant (Mann–Whitney test).

**Figure 3:**
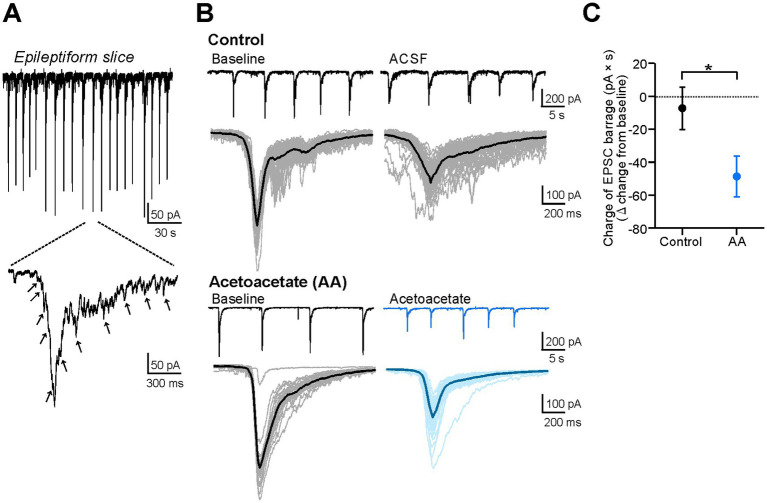
Acetoacetate reduces EPSC barrages in hippocampal pyramidal cells of epileptiform slices. **(A)** The barrages of EPSCs in epileptiform slices, measured by voltage-clamp recordings from CA1 pyramidal cells. Arrows indicate distinguishable EPSC inputs. **(B)** Epileptiform EPSC barrages in pyramidal cells, changed by a 20-min recording with no reagents for the control (*Control*) or by a 20-min application of 10 mM sodium acetoacetate (*Acetoacetate*). EPSC barrages were measured at a holding potential of −75 mV. Individual EPSC barrages for 5-min recordings were superimposed (*thin lines*) and averaged (*thick lines*). **(C)** Summary data from **(B)**, showing the changes in the charges of averaged EPSC barrages from the baseline in the control group (*n* = 5) and acetoacetate-treated group (*n* = 5). The charges were calculated as the area of the averaged EPSC barrages. The EPSC barrages were evaluated from 5-min recordings at the baseline and 15–20 min after the application of acetoacetate. ^*^p < 0.05 (Mann–Whitney test).

Individual slices were transferred to a submerged recording chamber, and perfused with oxygenated ACSF at room temperature. To induce epileptiform activity in the hippocampus, the following reagents were included into ACSF: potassium channel blockers (10 mM TEA-Cl and 3 mM CsCl), a GABA_A_ receptor blocker (100 μM picrotoxin), and a GABA_B_ receptor blocker (1 μM CGP-55845). Pyramidal cells in the hippocampal CA1 region were visualized by using an infrared differential interference contrast microscope equipped with a camera. Membrane potentials (Figures 1, 2) and synaptic currents (Figure 3) in CA1 pyramidal cells were measured by whole-cell recordings using a patch-clamp amplifier. Series resistance was typically less than 20 MΩ. Liquid junction potentials were not corrected. Electrical signals were low-pass filtered at 3 kHz and digitized at 10 kHz using an analog-to-digital converter. After stable recordings were confirmed, 10 mM sodium acetoacetate were bath-applied for 20 min. Control recordings were performed using the same protocol without acetoacetate.

Membrane potentials in CA1 pyramidal cells (Figures 1, 2) were measured in whole-cell current-clamp recordings. Patch pipettes were filled with an intracellular solution (in mM): 130 K-methanesulfonate, 6 KCl, 10 HEPES, 2 EGTA, 4 Mg-ATP, 0.3 Na_3_-GTP, and 5 phosphocreatine-Na (pH 7.3 adjusted with KOH). Membrane potentials were adjusted to −70 mV in normal slices (Figure 1) and adjusted to −75 mV in epileptiform slices (Figure 2). These negative potentials were used to easily visualize epileptiform bursts by suppressing spontaneous action potentials. In normal slices (Figure 1), firing and intrinsic properties were examined by 500-ms current injections from holding potentials of −60 mV, before and after the application of acetoacetate. Input resistance and Ih sag were examined by injecting negative currents of −50 pA, and the number of spikes and afterhyperpolarization of the first spike were examined by injecting positive currents of +50 pA. In epileptiform slices (Figure 2), the number of epileptiform bursts was evaluated by counting slow-depolarizing potentials with >8 mV in amplitude, and the number of spikes in each epileptiform burst was evaluated by counting action potentials in individual epileptiform bursts and averaging their numbers for 5 min. Cells were discarded if the rate of bursts including spikes at the baseline was less than 80%.

The barrages of synaptic currents (EPSC barrages) in CA1 pyramidal cells (Figure 3) were measured in whole-cell voltage-clamp recordings. Patch pipettes were filled with an intracellular solution (in mM): 130 Cs-methanesulfonate, 5 NaCl, 10 HEPES, 2 Na_4_-BAPTA, 4 Mg-ATP, 5 QX314-Cl, and 0.2 CaCl_2_ (pH 7.3 adjusted with CsOH). EPSCs were measured at holding potentials of −75 mV. Barrages of synaptic inputs with >50 pA in amplitude were evaluated as EPSC barrages, and the charges of averaged EPSC barrages for 5 min were compared before and after the application of acetoacetate. Cells were discarded if the EPSC barrages at the baseline were small in amplitude, typically less than 200 pA. In our recording condition, the peak amplitude of EPSC barrages was reduced even in the control group [302.8 ± 54.8 pA in the baseline and 156.8 ± 37.7 pA in ACSF (15–20 min after the baseline with no reagents)], whereas the charge of EPSC barrages was not changed in the control group (calculated by the area of EPSC barrages; 57.7 ± 16.6 pA × s in the baseline and 50.4 ± 10.6 pA × s in ACSF) (*n* = 5, see Figure 3B), and therefore the charge of EPSC barrages was analyzed in Figure 3C.

Sodium acetoacetate was prepared by the hydrolysis of ethyl acetoacetate as previously described (Kadowaki et al., 2017), which was based on original studies (Krebs and Eggleston, 1945; Owen et al., 1973). Ethyl acetoacetate (2.6 mL), 2 N NaOH (10.2 mL), and water (7.2 mL) were mixed and hydrolyzed by an incubation at 40°C for 90 min. The solution was cooled to stop the reaction, neutralized to pH 7.0 with HCl, and then fully lyophilized by a freeze dryer. The sodium acetoacetate was stored at −25°C until before use.

### Data analysis

In this study, electrophysiological data were obtained from 32 pyramidal cells in hippocampal slices prepared from 24 mice. Data analyses were performed using Igor Pro 6 (WaveMetrics), and the changes in electrical parameters from the baseline were compared between the control groups and acetoacetate-treated groups. Summarized data were represented as mean ± SEM. Statistical analyses were performed using SigmaPlot 12 (Systat Software), and the statistical significance was evaluated by non-parametric Mann–Whitney test for two group comparisons.

## Results

Previous studies have shown that ketone bodies exert weak or no effects on electrical activity in hippocampal slices (reviewed in Kawamura et al., 2016). To confirm this, we first examined the effects of acetoacetate (10 mM) on the membrane potentials of CA1 pyramidal cells in hippocampal slices from normal mice (Figure 1). This concentration of acetoacetate was selected because the ketogenic diet increases plasma ketone bodies at ~8 mM in rodents *in vivo* (Bough et al., 1999), and therefore, 2–10 mM ketone bodies have been used for *in vitro* electrophysiology of hippocampal slices (Thio et al., 2000; Ma et al., 2007; Juge et al., 2010; Samoilova et al., 2010; Kimura et al., 2012; Youssef, 2015; Kadowaki et al., 2017). We found that the membrane potentials of pyramidal cells were not affected by the application of acetoacetate for 20 min (Figure 1A). The changes in membrane potentials in the control group (−4.0 ± 0.4 mV from baseline, *n* = 5) were not significantly different from those in the acetoacetate-treated group (−3.0 ± 2.3 mV from baseline, *n* = 5) (Figure 1C; *p* = 0.15, Mann–Whitney test). The intrinsic membrane properties of pyramidal cells were not also affected by the application of acetoacetate for 20 min (Figure 1B). The changes in input resistance (*p* = 0.84, Mann–Whitney test), Ih sag (*p* = 1.00, Mann–Whitney test), the number of spikes (*p* = 0.42, Mann–Whitney test), and afterhyperpolarization (*p* = 0.84, Mann–Whitney test) were not significantly different between in the control group (*n* = 5) and in acetoacetate-treated group (*n* = 5) (Figure 1D). These results show that, consistent with previous studies, pyramidal cells in normal hippocampal slices were not electrically changed by even a high concentration of acetoacetate.

We then explored what kinds of conditions are required for acetoacetate to change the membrane potentials of CA1 pyramidal cells (Figure 2). To address this issue, we hypothesized that, although acetoacetate exerted no effects in normal hippocampal slices (Figure 1), it could change the membrane potentials of pyramidal cells in acutely-induced epileptiform slices. This hypothesis was inspired from the following two previous studies; one study shows that a long-term 2-week exposure to ketone bodies reduces spontaneous epileptiform activity in the organotypic hippocampal slices of Kcna1-knockout seizure mice (Kim et al., 2015). The other study shows that a short-term exposure to acetoacetate hardly affects EPSCs in normal hippocampal slices, but remarkably reduces EPSCs in acutely-induced epileptiform slices (Kadowaki et al., 2017). We therefore examined the effects of acetoacetate on electrical activity in epileptiform slices, which were acutely induced by applying convulsant blocker cocktails to normal hippocampal slices.

In current-clamp recordings, pyramidal cells were silent in hippocampal slices from normal mice (*Normal slice* in Figure 2A), but exhibited spontaneous epileptiform bursts when hippocampal slices were treated for >20 min with potassium channel blockers (10 mM TEA-Cl and 3 mM CsCl), a GABA_A_ receptor blocker (100 μM picrotoxin), and a GABA_B_ receptor blocker (1 μM CGP-55845) (*Epileptiform slice* in Figure 2A). We found that the epileptiform bursts were markedly weakened by the application of 10 mM acetoacetate for 20 min (Figure 2B). Further analyses revealed that the number of epileptiform bursts for 5 min was not significantly changed (−6.5 ± 6.7 from the baseline in the control group, *n* = 6; 20.0 ± 11.2 from the baseline in the acetoacetate-treated group, *n* = 6; *p* = 0.13, Mann–Whitney test), whereas the number of spikes in each epileptiform burst was significantly decreased (1.13 ± 0.41 from the baseline in the control group, *n* = 6; −2.48 ± 0.83 from the baseline in the acetoacetate-treated group, *n* = 6; *p* = 0.004, Mann–Whitney test) (Figure 2C). These results show that pyramidal cells in epileptiform hippocampal slices were electrically changed by the short exposure to acetoacetate, and also that acetoacetate weakened individual epileptiform bursts, but did not change the frequency of epileptiform bursts.

Seizures are characterized by hypersynchronous electrical activity. However, it remains unclear whether the weakening of epileptiform bursts by acetoacetate (Figure 2) is derived from the changes in synchronous synaptic inputs or the changes in intrinsic neuronal activity. To address this issue, we examined the effects of acetoacetate on synchronous synaptic inputs during epileptiform activity (Figure 3). In voltage-clamp recordings, the barrages of EPSCs were observed in the pyramidal cells of epileptiform slices (Figure 3A). The EPSC barrages are synchronous synaptic inputs that elicit epileptiform bursts. We found that the epileptiform EPSC barrages were reduced by the application of 10 mM acetoacetate for 20 min (Figure 3B). Quantitative analyses revealed that the charges of epileptiform EPSC barrages were significantly decreased by acetoacetate (−7.3 ± 12.9 pA × s from the baseline in the control group, *n* = 5; −48.7 ± 12.3 pA × s from the baseline in the acetoacetate-treated group, *n* = 5; *p* = 0.016, Mann–Whitney test) (Figure 3C). These results show that acetoacetate weakened epileptiform bursts by reducing synchronous synaptic inputs.

## Discussion

In the present study, we found that a short exposure to acetoacetate did not affect intrinsic electrical properties in the pyramidal cells of normal hippocampal slices (Figure 1), but weakened epileptiform bursts in the pyramidal cells of acutely-induced epileptiform slices (Figures 2, 3). Further analyses revealed that acetoacetate did not change the frequency of epileptiform bursts, but weakened individual epileptiform bursts by reducing synchronous synaptic inputs. Taken together, these results show that acetoacetate preferentially acts on epileptiform slices, which strongly reinforce previous studies (Kim et al., 2015; Kadowaki et al., 2017).

Ketone bodies, β-hydroxybutyrate and acetoacetate, are a hallmark of metabolic changes induced by the ketogenic diet, which suppresses epileptic seizures (reviewed in Rho, 2017). However, previous studies have shown that ketone bodies exert weak or no effects on normal hippocampal slices. Several studies have shown that ketone bodies have no effects on EPSCs in normal slices (Thio et al., 2000; Kimura et al., 2012; Youssef, 2015). Although acetoacetate is a strong inhibitor of VGLUTs (IC_50_ = 200 μM), even a high concentration of 10 mM acetoacetate reduced miniature EPSCs by only 25% in hippocampal slices (Juge et al., 2010). This discrepancy between *in vivo* seizure models and *in vitro* normal slices is presumably because normal slices do not reflect *in vivo* conditions (reviewed in Kawamura et al., 2016). In fact, previous studies have shown that no electrical changes are observed in hippocampal slices obtained from normal rodents fed the ketogenic diet, whereas EPSCs and epileptiform activity are reduced in hippocampal slices obtained from seizure models fed the ketogenic diet (kainate-induced seizure model in Stafstrom et al., 1999; Kv1.1α-knockout seizure model in Simeone et al., 2014). These studies indicate that the ketogenic diet has no actions on hippocampal slices from normal mice, but changes electrical parameters in those from seizure models.

Other studies have shown that ketone bodies themselves directly regulate electrical activity in epileptiform slices. One study has shown that a long-term exposure (2 weeks) to ketone bodies (5 mM β-hydroxybutyrate and 1 mM acetoacetate) reduces spontaneous epileptiform activity in hippocampal slices from Kcna1-knockout seizure models (Kim et al., 2015). Another study has provided more direct evidence, showing that a short exposure (15 min) to 10 mM acetoacetate reduces EPSCs in acutely-induced epileptiform slices made by applying convulsant blockers to normal slices, but does not change EPSCs in normal slices themselves (Kadowaki et al., 2017). The present study further reinforced this previous study; the same short exposure to acetoacetate weakened network-driven neuronal bursts in acutely-induced epileptiform slices (Figures 2, 3). Our patch-clamp recordings from single cells also revealed that acetoacetate attenuated individual epileptiform bursts, but did not affect the burst frequency (Figure 2). In addition, these *in vitro* effects of the short exposure to acetoacetate in epileptiform slices (Figures 2, 3) were consistent with previous studies showing the acute effects of acetoacetate on seizures *in vivo*, in which a single intraperitoneal injection of acetoacetate *in vivo* protects convulsion in audiogenic seizure-susceptible mice (Rho et al., 2002) and also reduces hippocampal seizures in a chronic model of temporal lobe epilepsy (Kadowaki et al., 2017).

There are some issues to remain unresolved in the present study. First, acetoacetate slightly increased the frequency of epileptiform bursts (see upper panel in Figure 2C). Although the underlying mechanisms remain unclear, a potential exploration is that acetoacetate attenuates epileptiform bursts, reduces voltage-dependent Ca^2+^ entry, and then weakens Ca^2+^-dependent afterhyperpolarization, which consequently shortens the repolarization phase and increases the burst frequency. If the individual bursts are weakened and asynchronized, seizure frequency *in vivo* might be decreased. Second, acetoacetate attenuated individual epileptiform bursts (Figure 2), but its molecular mechanisms remain unclear. Although K_ATP_ channels (Ma et al., 2007; Tanner et al., 2011), VGLUTs (Juge et al., 2010), and VDCCs (Kadowaki et al., 2017) are known to be the molecular targets of ketone bodies as electrical modulators, the most likely explanation at present is that acetoacetate reduces presynaptic glutamate release and decreases EPSC amplitude (Kadowaki et al., 2017), which consequently attenuates EPSC barrages (Figure 3). This is because acetoacetate increases paired-pulse ratio of EPSCs only in acutely-induced epileptiform slices, but not in normal slices (Kadowaki et al., 2017), which is closely similar with the present study. Although further studies will be required to clarify these unresolved issues, the present study provides strong evidence showing that ketone bodies preferentially act on hippocampal neurons under seizure conditions.

## Data Availability

The original contributions presented in the study are included in the article/supplementary material, further inquiries can be directed to the corresponding author.
